# Interpretable, Scalable, and Transferrable Functional Projection of Large-Scale Transcriptome Data Using Constrained Matrix Decomposition

**DOI:** 10.3389/fgene.2021.719099

**Published:** 2021-08-20

**Authors:** Nicholas Panchy, Kazuhide Watanabe, Tian Hong

**Affiliations:** ^1^Department of Biochemistry and Cellular and Molecular Biology, The University of Tennessee, Knoxville, Knoxville, TN, United States; ^2^RIKEN Center for Integrative Medical Sciences, Yokohama, Japan; ^3^National Institute for Mathematical and Biological Synthesis, Knoxville, TN, United States

**Keywords:** dimensionality reduction, gene set analysis, EMT, single-cell ‘omics, RNA-sequencing data

## Abstract

Large-scale transcriptome data, such as single-cell RNA-sequencing data, have provided unprecedented resources for studying biological processes at the systems level. Numerous dimensionality reduction methods have been developed to visualize and analyze these transcriptome data. In addition, several existing methods allow inference of functional variations among samples using gene sets with known biological functions. However, it remains challenging to analyze transcriptomes with reduced dimensions that are interpretable in terms of dimensions’ directionalities, transferrable to new data, and directly expose the contribution or association of individual genes. In this study, we used gene set non-negative principal component analysis (gsPCA) and non-negative matrix factorization (gsNMF) to analyze large-scale transcriptome datasets. We found that these methods provide low-dimensional information about the progression of biological processes in a quantitative manner, and their performances are comparable to existing functional variation analysis methods in terms of distinguishing multiple cell states and samples from multiple conditions. Remarkably, upon training with a subset of data, these methods allow predictions of locations in the functional space using data from experimental conditions that are not exposed to the models. Specifically, our models predicted the extent of progression and reversion for cells in the epithelial-mesenchymal transition (EMT) continuum. These methods revealed conserved EMT program among multiple types of single cells and tumor samples. Finally, we demonstrate this approach is broadly applicable to data and gene sets beyond EMT and provide several recommendations on the choice between the two linear methods and the optimal algorithmic parameters. Our methods show that simple constrained matrix decomposition can produce to low-dimensional information in functionally interpretable and transferrable space, and can be widely useful for analyzing large-scale transcriptome data.

## Introduction

Recent developments in RNA-sequencing technology have enabled the collection of large-scale transcriptome data at high speed. For example, single-cell RNA-sequencing (scRNA-seq) data of many biological systems have been accumulating rapidly and provide opportunities to gain insights into complex biological processes at both the systems level and the single-cell resolution. Together with the advances in experimental techniques, the recent development of computational methods, including those for dimensionality reduction, allow the visualization and analyses of high-dimensional transcriptome data in low-dimensional space. For example, *t*-distributed stochastic neighbor embedding (tSNE) and Uniform Manifold Approximation and Projection (UMAP) have been instrumental to tackling challenges in transcriptome data visualization and are widely used in biomedical research (Van der Maaten and Hinton, 2008; Stein-O’Brien et al., 2018; Becht et al., 2019; Luecken and Theis, 2019). However, dimensionality reduction methods usually do not provide low-dimensional space that is directly interpretable in terms of biological functions: while these approaches cluster related samples, the positioning of samples along the derived dimension may not correspond to the degree of any biological process even if a predefined gene set with similar functions is chosen before the reduction. In addition, the contribution or significance of individual genes related to the derived dimension cannot be accessed directly with these methods. The lack of interpretability of the dimensions makes it challenging to visualize and analyze the progression of the samples (cells) in known biologically functional space.

Existing methods for functional quantification, such as Z-score and Gene Set Variation Analysis (GSVA; Hänzelmann et al., 2013), are useful for obtaining “functional scores” with the expression levels of multiple genes involved in the same biological process. However, these methods do not have transferability in that the scoring systems obtained with one dataset cannot be used to analyze other datasets directly. This limits the utility of these methods in predicting the progress of new data points, and in studying the relationships between functional spaces in different experimental settings.

One example of cellular processes that contains crucial quantitative information is epithelial-mesenchymal transition (EMT). While extreme changes of cell fate and morphology occur in the classical form of EMT, recent studies with cancer and fibrosis showed that partial EMT involving intermediate states are prevalent, and it may be responsible for pathogenesis (Pastushenko et al., 2018). To quantify the degree of EMT in EMT-induced cell lines and tumor samples, several previous studies analyzed transcriptomic data and their projections onto epithelial (E) and mesenchymal (M) dimensions (Tan et al., 2014; George et al., 2017; Cursons et al., 2018; Chakraborty et al., 2020; Panchy et al., 2020; Hirway et al., 2021). Recently, scRNA-seq analysis has shown that the progression of EMT is highly dependent on inducing signals and cell types (Cook and Vanderhyden, 2020). However, it remains challenging to analyze rapidly accumulating transcriptome information on EMT for obtaining biological insights across multiple conditions. Improvement of methods for reducing dimensions of expression data in a functionally meaningful manner is necessary.

In this study, we used gene set filtered variants of both non-negative principal component analysis (gsPCA) and non-negative matrix factorization (gsNMF) to analyze progression of EMT in single cells at multiple timepoints. We show that these methods describe large-scale transcriptome data of multiple EMT stages in low-dimensional and functionally interpretable space. Taking advantage of the methods’ transferability, we constructed dimensionality reduction models that can predict the stages of EMT with data from timepoints that were not used for model construction. We show that these linear methods can be used to compare functional spaces across multiple experimental conditions. Furthermore, we demonstrate the utility of our approach in visualizing drug responses in heterogeneous single cell data. With a validation scheme for rigorous testing, we provide recommendations for the choice of the methods and the parametric settings. Overall, our work provides a new toolbox for analyzing large-scale transcriptome data with efficient visualization and functional quantification.

## Results

### Overview of Method and Performance Evaluation

**FIGURE 1:**
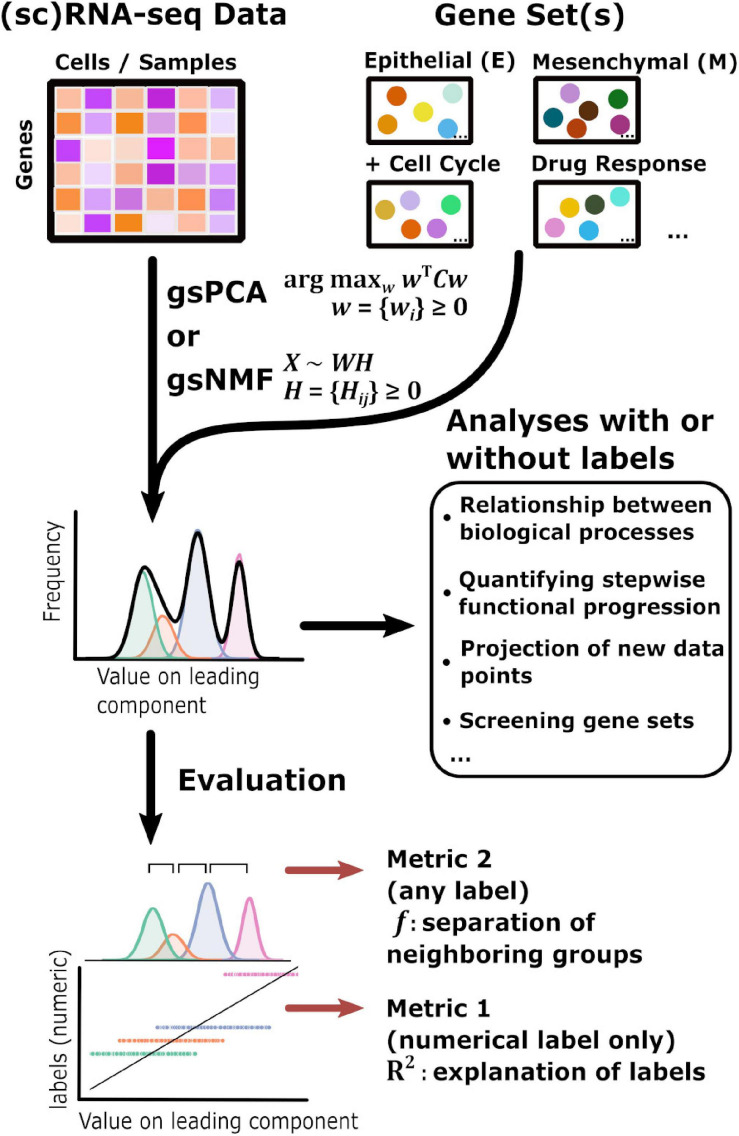
Schematic of the gene set non-negative matrix factorization (gsNMF)/gene set non-negative principal component analysis (gsPCA) analysis process. A diagram of the analysis process used in this study beginning with input data in the form of sequencing data and gene sets. gsNMF/gsPCA is applied to this data to generate a functional scoring or space in the form of component scores (see “Materials and Methods” section for details). These scores can be used in two ways. First, without further data labels, scores can be used to look at relationships between or across biological processes beginning with low-dimensional visualization to identify trends and putative groups. Quantities such as the correlation between different functional scores can be computed for analysis. In addition, the transferable nature of these models means that they can be used to infer the position of new data points and contributions of individual genes, which allows assessment of their importance. Screening can be done both between gene sets and within gene sets. Secondly, when data labels are present, different metrics can be used to assess the performance of a functional score in terms of capturing variance: the common language effect size or *f*-probability can be used to evaluate how well the functional score separates two distinct populations while the variance explained or *R*^2^ can evaluate how much of the variation of a numeric variable representing biological progression, such as time, that the functional score can explain across the data.

The overall goal of our method is to find low-dimensional space of transcriptome data that has both biologically meaningful directionality and the ability to represent data points not used in the procedure to derive the space. This requires one or more preselected functional gene sets, which are readily available in publicly available databases such as Molecular Signature Database (Liberzon et al., 2011), and can be defined manually (Figure 1). We propose two linear approaches of matrix decomposition: gsPCA and gsNMF (see “Materials and Methods” section for details). Briefly, gsPCA finds the optimal component (projection) by maximizing the variance of the projected data points under the constraint that each functional gene has a non-negative loading value. For gsNMF, the gene-set-filtered transcriptome matrix is approximated by the product of two non-negative matrices, one of which represents a “meta” expression profile across samples, while the other represents the non-negative coefficients of the functional genes (the procedure for obtaining the number of components is described in Supplemental Methods). Following gsNMF, the leading component is selected for subsequent analyses (see “Materials and Methods” section). With either gsPCA or gsNMF, transcriptome data can be projected onto an axis whose direction unambiguously represents expression of the gene set and can be interrogated to reveal the contribution or association of individual genes in the set to scores along the axis.

To test the performance of gsPCA and gsNMF in capturing biological progression through functional space, we first used time-course datasets containing single cells treated with EMT-inducing signals for various periods of time (Cook and Vanderhyden, 2020). In addition to the biological importance of the stepwise progression in EMT (Pastushenko et al., 2018; Kröger et al., 2019), the time labels in the datasets allow us to evaluate the performance of the functional projection. Specifically, we used two metrics for the evaluation: the coefficient of determination (*R*^2^) for quantifying how well the projected values explain the time labels, and the common language effect size (*f*) for measuring the separation between two neighboring subsets of data with two labels (McGraw and Wong, 1992; See “Materials and Methods” section). The usage of *R*^2^ is only possible when the labels are numerical, while *f* can be used with any type of label (Figure 1). Note that our overall goal is not clustering the data points. Instead, we aim to represent the progression along biologically meaningful axes. In addition, neither gsPCA nor gsNMF requires data labels for analysis. The two metrics are only used for evaluation. In later sections, we will show analyses with additional data sets in which labels are categorical and the biological processes are non-EMT.

### gsPCA and gsNMF Capture Cell State Progression in Low Dimensional Functional Space

**FIGURE 2:**
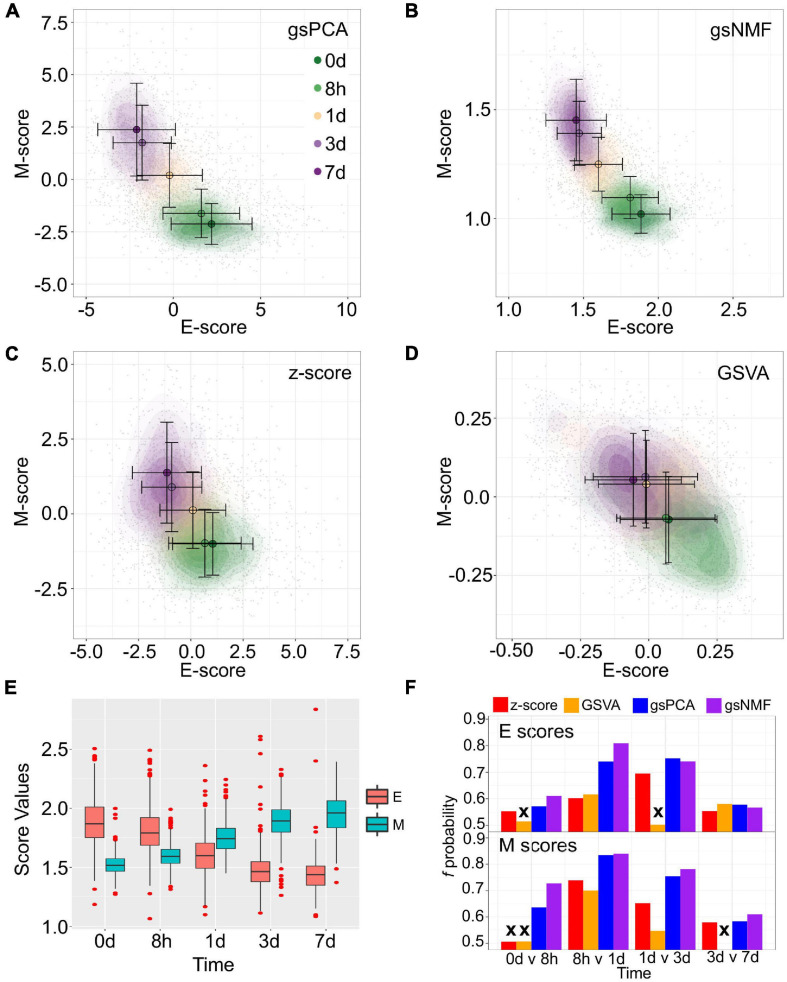
Visualization of epithelial-mesenchymal transition (EMT) progression in TGF-β induced A549 cells by multiple scoring methods. **(A–D)** Contour plots of gene set scores of E (X-axis) and M (Y-axis) genes from four different scoring methods, gsPCA **(A)**, gsNMF **(B)**, z-score **(C)**, and GSVA **(D)**. Color indicates the time of TGF-β induction from 0 days (dark green) to 7 days (dark purple). Circles indicate the mean E- and M-score of samples from each time point and the associated error bars show the standard deviation. **(E)** A box-plot showing the distribution of E (red) and M (blue) scores across all five time points of TGF-β induction from the gsNMF model. Whiskers indicate the 1.5 inter-quartile range of each distribution while the red points indicate outliers beyond this range. **(F)** Bar chart of the *f* probability values for E (top) and M (bottom) scores between all consecutive pairs of time points. Color indicates the method used to produce the score: red is z-score, orange is GSVA, blue is gsPCA, and purple is gsNMF. Bars marked by an “x” indicates that the score did not significantly separate the samples from those time points (Mann–Whitney *U*-test, *p* < 0.05).

To show the performance of the proposed methods, we first used two signature gene sets whose high expressions represent the epithelial (E) and mesenchymal (M) states, respectively (Tan et al., 2014; Watanabe et al., 2019; Panchy et al., 2020). With the E and M gene sets, we first performed gsPCA and gsNMF on time-course single-cell transcriptomes of TGF-β-treated A549 cells using two components per model for each gene set (Cook and Vanderhyden, 2020). The two gene sets contain 179 and 114 genes, respectively, in the A549 data set. We then projected the single-cell data from the first five time points, which represent continuous EMT progression, onto the leading dimension for each gene set. This produced two-dimensional plots with dimensions that can be viewed as the progression of cell states in the epithelial and the mesenchymal spectrums (Figures 2A,B). We then compared the performance to two widely used approaches: Z-score and GSVA (Figures 2C,D). We found that gsPCA and gsNMF both better explained the overall variance of time across the first five time points of EMT progression (Adjusted *R*^2^ = 0.46 and 0.48, respectively) than Z-score (Adjusted *R*^2^ = 0.31) and GSVA (Adjusted *R*^2^ = 0.08). Likewise, when considering neighboring time points, we found that E-scores tended to decrease and M-scores tended to increase with time of TGF-β treatment (Figure 2E), with both scores significantly separating all neighboring time points for gsPCA and gsNMF and yielding higher *f* probabilities than other methods in all but one case (E-scores at 3 vs. 7 days, Figure 2F). This suggests that gsPCA and gsNMF not only serve as visualization methods of functional space with defined gene sets, but also describe heterogeneous cell populations containing transitional information in a rigorous fashion. Between the two methods, we found the gsNMF performed better with regard to both overall variance (Adjusted *R*^2^ 0.48 vs. 0.46) and separating time points (Figure 2F) than gsPCA. However, gsNMF requires selecting the leading dimension based directly on correlation with time of EMT progression, suggesting that gsPCA may be more reliable in a purely unsupervised setting (see “Materials and Methods” section).

In the next few sections, we show various utilities of these linear methods based on their transferability and high-performance features. Because gsNMF gives the best performance with the A549 EMT data set, our discussion will focus on results obtained with gsNMF. The results using gsPCA, which had similar performance in all cases, are included in Supplementary Materials.

### Prediction of Cell States With Data From New Conditions

**FIGURE 3:**
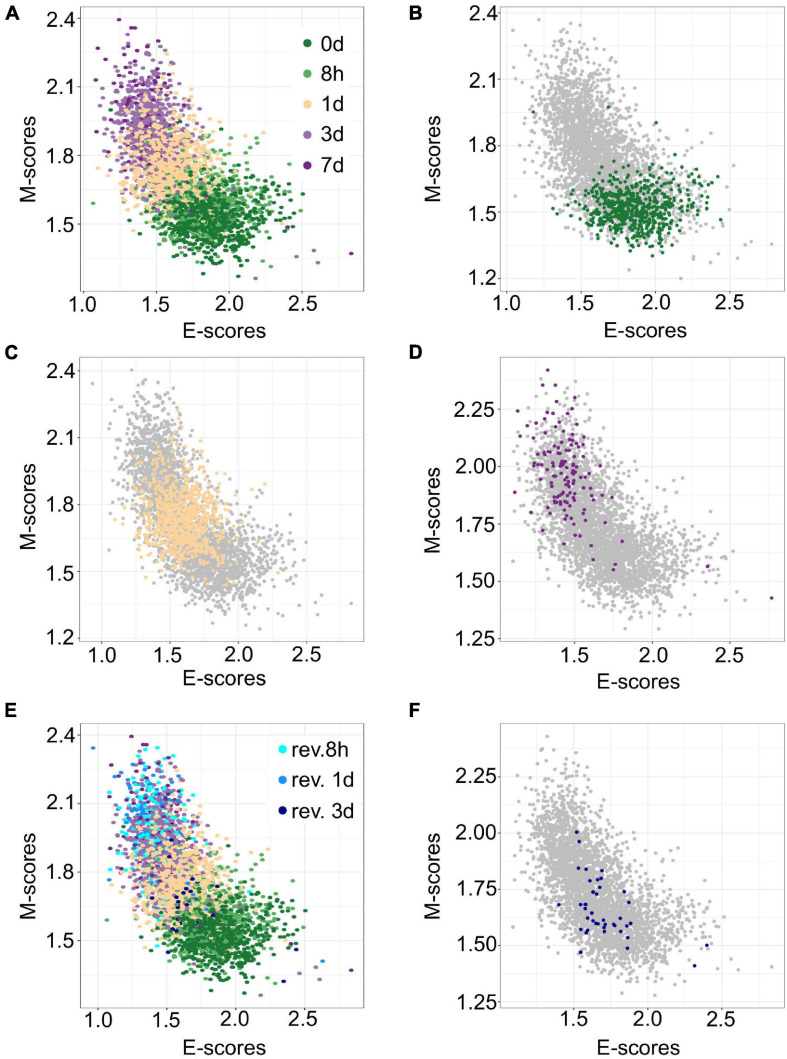
Predicting A549 samples from specific time points using gsNMF. **(A)** Scatter plot of E (X-axis) and M (Y-axis) scores for all TGF-β induction samples using gsNMF. Samples from different time points are indicated by color going from 0 days (dark green) to 7 days (dark purple). **(B–D)** Scatter plot of 0-day (green, **B**), 1-day (yellow, **C**), and 7-day samples (purple, **D**) inferred using a gsNMF model built with all other time points (gray). **(E)** A scatter plot of TGF-β induction samples with TGF-β reversion samples (i.e., 7 days induction followed by removal from TGF-β). Induction samples are labeled as in panel **(A)**, while reversion samples are colored blue, with darker shade indicating longer time since removal. **(F)** Scatter plot of 3-day reversion samples (dark blue) inferred using a gsNMF model built with all non-reversion time points (gray).

The transferability of gsPCA and gsNMF methods allows the projection of new high-dimensional data points onto previously derived functional dimensions. Similarly, these methods can be used to derive functional dimensions with partial information of the biological process in terms of its stages. To show the predictive power of gsNMF, we removed samples from the 0-, 1-, and 7-day (including revertant) time points in the A549 EMT data (i.e., the start, middle, and the end of the continuous portion of TGF-β induction) and then performed the dimensionality reduction. We found that the low-dimensional functional space was robust with respect to the removal, regardless of whether the missing time point is in the middle of the progress or at the extremes (Figures 3A–D), such that when we projected the removed data points onto the space derived from a partial dataset, their positions were highly correlated with their positions when they were included in the data set [Pearson correlation coefficient (PCC > 0.95)]. However, while the inferred 1-day samples were similarly separable from samples in 8-h (*f* = 0.81 for E, 0.84 for M) and 3-day (*f* = 0.75 for E, 0.79 for M) time points, we observed reduced separability between the both inferred 0-day vs. 8-h (*f* = 0.52 for E, 0.63 for M) and 3-day vs. inferred 7-day (*f* = 0.53 for E, 0.55 for M) time points, with E-scores not significantly separating the first and the last time points (Mann–Whitney *U*-test, *p* = 0.13 and 0.16, respectively). We also applied the same inference procedure to samples which were exposed to a transient EMT-inducing signal and allowed to revert. However, because the 8- and 24-h reversion samples largely overlap with 7-day (hence their removal for 7-day inference, Figure 3E), we focused on inferring 3-day reversion samples after performing dimensionality reduction on the data set without any reversion samples. We found that 3-day reversion samples were positioned in the middle of the EMT spectrum, consistent with when they were included in functional space construction (PCC = 0.99 for E and 0.98 for M, Figure 3F). Additionally, the inferred 3-day reversion samples were similarly separable from the 7-day samples (*f* = 0.91 for E, 0.91 for M) as when they were when included in functional space construction (*f* = 0.90 for M, 0.91 for M). We obtained similar results using gsPCA when inferring the position of samples from missing time points (Supplementary Figure 1), but neither E- nor M-scores significantly separated the end points (0-day vs. 8-h and 3- vs. 7 day). These results suggest that gsPCA and gsNMF can predict cell states of new data without retraining the model, and that these methods can be used to predict new cell states that have not been observed directly, though it may be difficult to separate these samples when they are positioned the edge of the spectrum and/or when the new samples are closely related to existing samples.

### Using Functional Space Across Cell Lines

**FIGURE 4:**
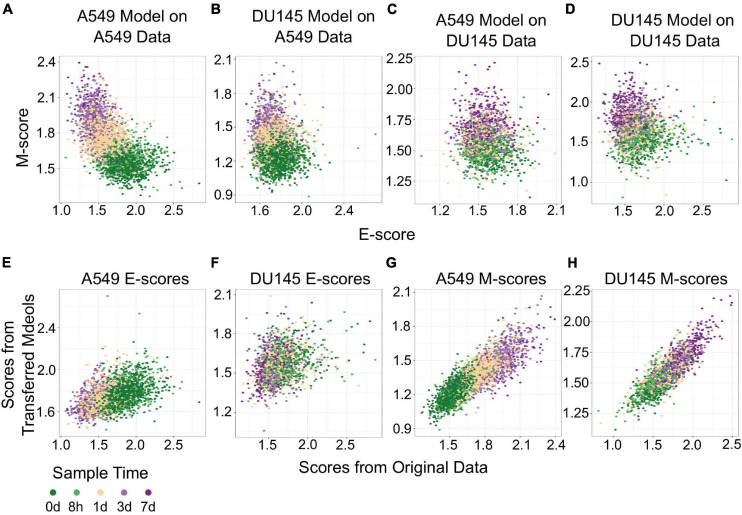
Transferring gsNMF models between A549 and DU145 TGF-β induced samples. **(A–D)** Scatter plot of E (X-axis) and M (Y-axis) scores for different combinations of data and gsNMF model: **(A)** A549 model on A549 data, **(B)** DU145 model on A549 data, **(C)** A549 model on DU145 data, and **(D)** DU145 model on DU145 data. Samples from different time points are indicated by color going from 0 days (dark green) to 7 days (dark purple). **(E,F)** Comparison of E-scores of samples from A549 **(E)** and DU145 **(F)** data. The X-axis is the E-score from using the model from the same data set (A549 on A549 and DU145 by DU145), while the Y-axis is the E-score from the opposite model (DU145 on A549 and A549 on DU145). Samples from different time points are indicated by color going from 0 days (dark green) to 7 days (dark purple). **(G,H)** Comparison of M-scores of samples from A549 **(G)** and DU145 **(H)** data. The X-axis is the M-score from using the model from the same data set (A549 on A549 and DU145 by DU145), while the Y-axis is the M-score from the opposite model (DU145 on A549 and A549 on DU145). Samples from different time points are indicated by color going from 0 days (dark green) to 7 days (dark purple).

The transferability of gsNMF can be extended to data from different cell lines. We performed gsNMF on single-cell transcriptomes of TGF-β-treated DU145 from Cook and Vanderhyden (2020) using the same procedure as A549 and obtained a moderate explanation of variance in time of EMT progression using the E and M dimensions (Adjusted *R*^2^ = 0.31). We then inferred the position of the five continuous time points in the A549 data set using the DU145 model and vice versa (Figures 4A–D). Transferred models (DU145 on A549 and A549 on DU145) were able to separate the individual time points, but overall performance decreased as they can explain only part of the variance seen in the original models (Adjusted *R*^2^ = 0.30 for DU145 on A549 and 0.25 for A549 on DU145). Therefore, it was expected that the individual sample scores would be positively correlated between models along both the E (Figures 4E,F) and M dimensions (Figures 4G,H). However, while the correlations between all pairs of scores were significant (minimum *p* = 2.7e−73), the correlation between E-scores was weaker overall and worse for models of DU145 (PCC = 0.31) than models of A549 (PCC = 0.46). Comparably, the M-scores for both models of A549 (PCC = 0.84) and models of DU145 (PCC = 0.84) were more highly correlated and consistent between models. However, none of the sample scores between A549 and DU145 models were as correlated as inferred sample scores from missing point and the complete A549 model (PCC > 0.95). This suggests a reduced transferability across cells lines compared to within cells lines. In addition, across the data sets we used, changes along the M dimension were more consistent than the E dimension. We observed similar results using gsPCA, including M-scores being more correlated (PCC, A549 = 0.92, DU145 = 0.94) than E-scores (PCC, A549 = 0.76, DU145 = 0.72; Supplementary Figure 2). This is consistent with the fact that the same inducing agent was used across all cell lines, and also implies that inducing EMT in different cell types may yield more consistent changes in M genes compared to E genes.

### Using Functional Space Across Experimental Conditions

**FIGURE 5:**
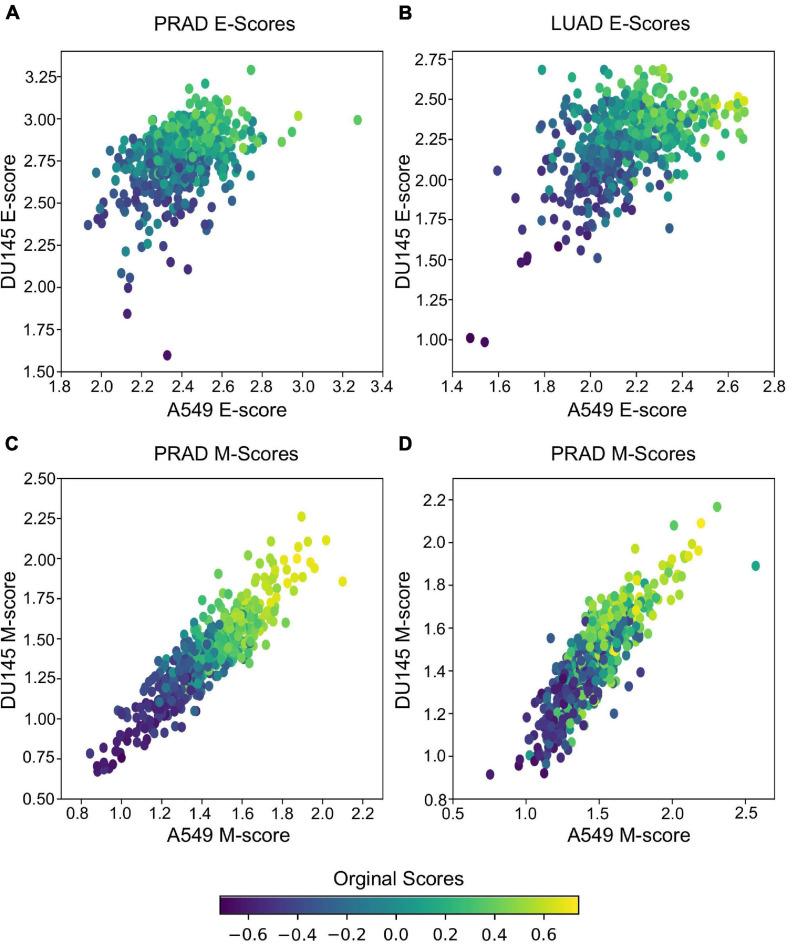
Transferring gsNMF models to TCGA data. **(A,B)** Scatter plots of E-scores for PRAD **(A)** and LUAD **(B)** from transferring gsNMF models built on A549 (X-axis) and DU145 (Y-axis) data. The color of individual points indicates the original GSVA based E-score of the TCGA data set. **(C,D)** Scatter plots of M-scores for PRAD **(C)** and LUAD **(D)** from transferring gsNMF models built on A549 (X-axis) and DU145 (Y-axis) data. The color of individual points indicates the original GSVA based M-score of the TCGA data set.

In addition to predicting the locations in the functional space across cell lines, gsPCA and gsNMF can be used across both experimental conditions and cell types. To test the cross-condition transferability, we first used our low-dimensional functional EMT space for A549 and DU145 cells to analyze tumor transcriptomes measured with bulk RNA-seq (The Cancer Genome Atlas, TCGA). To perform the most comparable transfer, we used lung adenocarcinoma (LUAD) and prostate adenocarcinoma (PRAD) data, which correspond to A549 and DU145 in terms of tissue type. We considered transfers between both similar (projecting LUAD data by a A549-trained model, and PRAD by a DU145-trained model) and dissimilar (LUAD by DU145 and PRAD by A549) cell types. We found that the low-dimensional functional space obtained with in-vitro data captured tumor sample heterogeneity in the EMT spectrum when compared to our previous GSVA analysis of the same data (Figure 5). Overall, the original E- and M-scores were significantly correlated with the A549 models in all cases (smallest *p* = 2.8e−34). Models from both cell lines showed similar correlation with the original GSVA scores, except in the case of PRAD scores, where the DU145 model was better correlated than the one built on A549 data (Table 1). We also observed that M-models built on A549 and DU145 data were more similar to each other than E-models, and we obtained similar results with gsPCA (Supplementary Figure 3), which showed greater overall correlation with GSVA scores, but the same pattern of reduced correlation for the A549 model of PRAD E-scores (Supplementary Table 1).

**TABLE 1:** Pearson correlation coefficients of E and M scores between GSVA, A549, and DU145 models of TCGA data.

TCGA data set	GSVA vs. A549	GSVA vs. DU145	A549 vs. DU145
PRAD E-genes	0.49	0.72	0.43
LUAD E-genes	0.72	0.71	0.52
PRAD M-genes	0.85	0.84	0.90
LUAD M-genes	0.62	0.65	0.86

It should be noted that these results are partly due to higher average correlation of expression of EMT genes in bulk RNA-seq data (average PCC = 0.28 LUAD, 0.38 PRAD for all pairs of M-genes, average PCC = 0.18 LUAD, 0.10 PRAD for E-genes), compared to the scRNA-seq data (average PCC = 0.01 in all cases). This is expected given that bulk RNA-seq is derived from populations rather than individual cells, but as a result, the effect of differentially weighing individual genes across models and components within models is reduced. This would explain the stronger correlation of M-scores between A549 and DU145 derived models, as well as the reduced performance of A549 on PRAD E-gene data, which is the most variable bulk RNA-seq data set. Yet, as the same time, this would suggest the variance present in PRAD bulk RNA-seq data is more similar to the model built on DU145 scRNA-seq data, than scRNA-seq data from a more dissimilar background. This also has implication for comparing multiple model components as they tend to be more similar in the bulk RNA-seq model despite if they were anti-correlated (gsNMF) or relatively uncorrelated (gsPCA) in the original scRNA-seq model or other scRNA-seq data (see Supplementary Table 2). Nonetheless, we have shown that the transferred models are, overall, consistent with the prior analysis of TCGA data and detected the expected variance in bulk RNA-seq data when it is present.

### Using Functional Space Across Spatial and Temporal Progression

**FIGURE 6:**
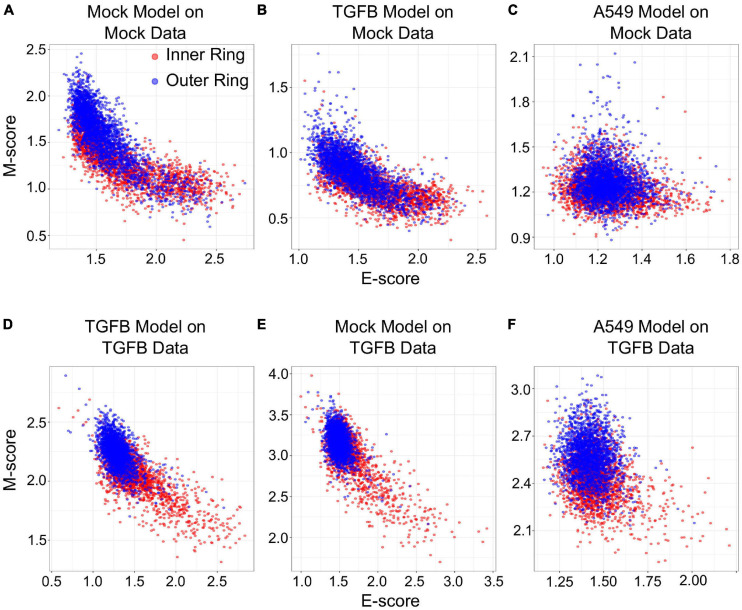
Transferring gsNMF models between temporal and spatial data sets. **(A–C)** Scatter plots of E (X-axis) and M (Y-axis) scores for Mock spatial data from gsNMF models built on different data sets: Mock spatial data **(A)**, TGF-β induced spatial data **(B)**, and TGF-β induced A549 temporal data **(C)**. The color of the sample indicates whether it originates from a cell in the inner-ring (non-motile, red) or the outer ring (motile, blue). **(D–F)** Scatter plots of E (X-axis) and M (Y-axis) scores for TGF-β spatial data from gsNMF models built on different data sets: TGF-β induced spatial data **(D)**, Mock spatial data **(E)**, and TGF-β induced A549 temporal data **(F)**. The color of the sample indicates whether it originates from a cell in the inner-ring (non-motile, red) or the outer ring (motile, blue).

We next examined if gsNMF can produce transferrable models that reveal both spatial and temporal progression of EMT. Using single-cell RNA-seq, McFaline-Figueroa et al. (2019) previously found that epithelial cells exhibit an E to M spectrum from the inner position of a colony to the outer position. This dataset that contains binarized identities (inner and outer) obtained with macro-dissection (defined as spatial EMT data) from two experiments, one in which cells were allowed to migrate without external induction of EMT (Mock), and one in which EMT was induced with TGF-β (TGF-β). Since there are only two populations in this data set, the leading dimension for E- and M-scores was chosen to maximize the separation based on the *f* probability. Overall, three analyses were performed for each data set: spatial data with its own gsNMF model, spatial data with the model from the other spatial data set (TGF-β on Mock and Mock on TGF-β), and spatial data with A549 time series model (Figure 6). As with our previous results, the best separation of inner and outer data points was observed when Mock (*f* = 0.61 for E, 0.73 for M) and TGF-β (*f* = 0.77 for E, 0.82 for M) data sets had their own model applied to them. However, for Mock data, the TGF-β model (*f* = 0.64 for E, 0.69 for M) outperformed the A549 model (*f* = 0.45 for E, 0.60 for M) on both dimensions and, in fact, the E dimension of the A549 model did not effectively separate inner and outer points in the Mock data (*p* = 0.99). In comparison, the Mock model better separated TGF-β inner and out points in the E direction (*f* = 0.68 for E, 0.63 for M), while the A549 model better separated them in the M direction (*f* = 0.59 for E, 0.76 for M). gsPCA models gave similar results, including the A549 model yielding better performance along the M-dimension (*f* = 0.76) for TGF-β data than Mock data (*f* = 0.68; Supplementary Figure 4).

The fact the A549 model better separated TGF-β spatial points along the M dimension than the Mock model, but did not outperform TGF-β on the Mock model suggests that there is conserved TGF-β induced M-gene expression regardless of context. To explore the basis of this similarity in M-scores, we compared the coefficient matrices (*H*, see “Materials and Methods” section) between Mock, TGF-β, and A549 gsNMF models, which represent the weights of individual genes along the components. We found little correlation between A549 and spatial E-gene coefficient values for the lead dimension (PCC = −0.02. *p* = 0.82 for Mock; PCC = 0.06, *p* = 0.57 for TGF-β), however, while there was also little correlation between A549 and spatial M-gene coefficient values for the Mock model (PCC = 0.02, *p* = 0.83) there was significantly positive correlation for the TGF-β model (PCC = 0.48, *p* = 8.8e–7). Additionally, we examined which genes were in the top 10th percentile of coefficient values across models and found that the A549 and TGF-β models share six M-genes (FN1, LGALS1, SERPINE1, TAGLN, TPM2, and VIM), compared to three E-genes (ELF3, PERP, and SLPI). Furthermore, another four E-genes (AREG, KRT18, KRT8, and NQO1) were in the top 10th percentile of A549 E-gene coefficient values, but the bottom 10th percentile of TGF-β E-gene coefficient values. We observed similar results from gsPCA, finding significant correlation of loading values only between A549 and TGF-β M-models (PCC = 0.61, *p* = 5.8e–11) with many of the same genes in the top 10th percentiles of both models (FN1, TPM2, VIM, TAGLN, GLIPR1, and LGALS1). Notably, the M-genes with high coefficient values in both A549 and TGF-β models across both A549 and TGF-β models are key regulators/inducers of EMT (FN1, LGALS1, and VIM; Mendez et al., 2010; Griggs et al., 2017; Zhu et al., 2019) or specific activators of migratory behavior in epithelial/cancer cells (TAGLN, TPM2; Lee et al., 2010; Shin et al., 2017). Conversely, while KRT8 and KRT18 are considered epithelial cytokeratins (Tomaskovic-Crook et al., 2009), both of these genes undergo an initial increase in expression in the A549 time-course (Supplementary Figure 5), compared to largely unaltered distributions across the inner and outer samples of migration data. This is consistent with previous observations that, both KRT8 (Wang et al., 2020) and KRT18 (Zhang et al., 2019) are over-expressed/aberrantly expressed in certain human cancers and such expression is associated with cancer progression/poor-prognosis. This potentially reflects intermediate EMT states caused by full or partial arrest of the process at an early timepoint, independent of the resulting migratory potential of the cells. Coefficient values for all genes in each model can be found in Supplementary Table 3.

Together, these results suggest a coherence of the progression of the EMT program in both the spatial and temporal context with regard to M-genes, while E-gene progression appears to be more sensitive to context, being only transferable between the two spatial data sets. The coefficient values of genes across both contexts offers insight into the difference in transferability between E and M models: high scoring M-genes across both contexts constitute important drivers of EMT/migration, suggesting common regulatory mechanisms, while the differential expression of KRT8 and KRT18 across time, but not space, suggests E-gene expression can be sensitive to biological context. Finally, these results highlight the usefulness of the transferability of gsPCA and gsNMF outside of a time series context, where performance may need to be evaluated on discrete groups.

### Characterizing Relationships Among Multiple Functional Spectrums

To test the capacity of gsNMF to infer functional spaces across a broader range of gene sets and data, we first returned to the A549 data set and examined the expression changes of multiple gene sets across EMT progression. Taking advantage of the high-efficiency of this method, we began with 5455 C2 curated gene sets from the Molecular Signature Database (see “Materials and Methods” section) and applied a gsNMF model to A549 data for each. For simplicity, we used a two-component model, but we applied stricter convergence and selection criteria because of the diversity of gene set size and coverage by the data set (see “Materials and Methods” section). Overall, 867 gene sets (15.9%) had a leading dimension whose magnitude of correlation (PCC) was > 0.5 (Supplementary Table 4). As such, we expected that functional spaces constructed from highly correlated gene sets should show similar results to our original E vs. M functional space.

**FIGURE 7:**
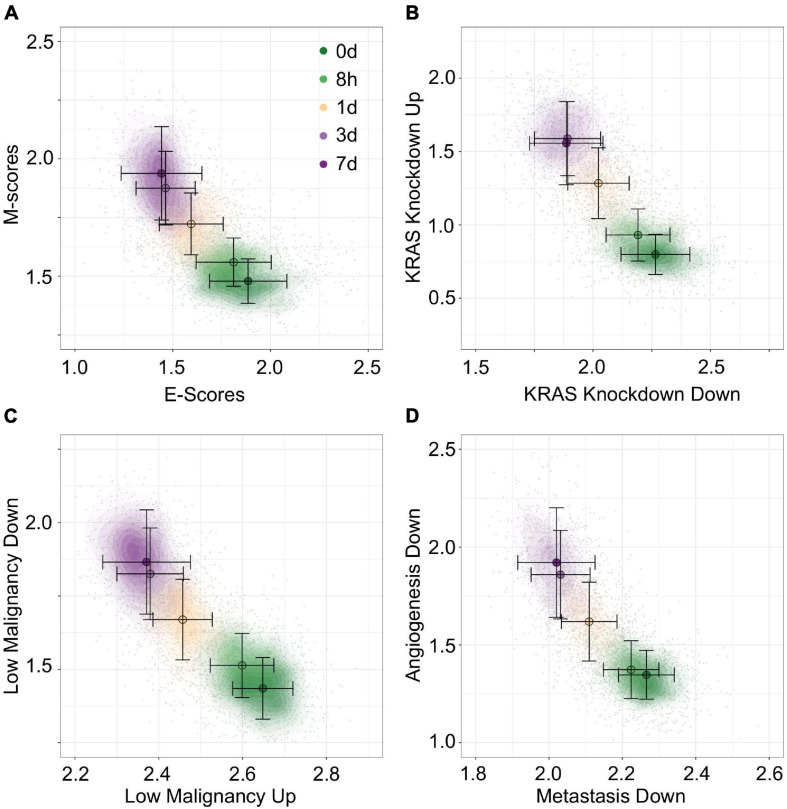
Visualization of EMT progression in TGF-β induced A549 cells by multiple gene sets. **(A–D)** Contour plots of A549 functional space generated using gsNMF with different gene sets: E vs. M **(A)**, KRAS knockdown up and down **(B)**, non-malignant ovarian cancer up and down **(C)**, and metastasis downregulation vs. angiogenesis downregulation **(D)**. Color indicates the time of TGF-β induction from 0 days (dark green) to 7 days (dark purple). Circles indicate the mean gene set score of samples from each time point and the associated error bars show the standard deviation.

To construct unambiguous functional spaces, we initially focused on pairs of up/down regulated gene sets where the leading dimensions had a high magnitude of correlation (PCC), but opposite sign, in order to emulate our original E/M model of EMT progression for A549 (Figure 7A). For example, two pairs of gene sets, up regulation or down regulation in response to KRAS knockdown (SWEET_KRAS_TARGETS, Figure 7B) and up regulation or down regulation in low-malignancy ovarian cancer relative to control (WAMUNYOKOLI_OVARIAN_CANCER_LMP, Figure 7C), yielded functional spaces similar to E and M genes (Figure 7A) and captured a similar amount of variance explained among non-revertant cells (*R*^2^ = 0.48 and 0.49, respectively). Furthermore, the results suggest that EMT progression is correlated with expression of genes normally repressed by KRAS, a pro-proliferation signal, and anti-correlated with the expression of genes associated with tumorigenic, but non-metastatic ovarian cancer, consistent with the idea of the E state of EMT being pro-proliferative and the M state being pro-migratory. However, not all pairs of gene sets provide well defined functional spaces: for example, the gene set down-regulated in metastatic vs. non-metastatic head and neck tumors (RICKMAN_METASTASIS_DN) produced a strong anti-correlated leading dimension (PCC = −0.63), but the leading dimension of the up-regulated variant has a far smaller magnitude of correlation (PCC = 0.34). However, combining the metastatic down-regulated gene set with another correlated gene set, genes silenced during angiogenesis (HELLEBREKERS_SILENCED_DURING_TUMOR_ANGIOGE NESIS, PCC = 0.66), generated a functional space of EMT progression competitive with E and M genes (Figure 7D, *R*^2^ = 0.48). As such, functional space constructs need not be confined to reciprocal or connected gene sets, though this does not excluded the possibility of an underlying, common genetic basis between these functional spaces. Nevertheless, the divergent origins of the gene sets in terms of the biological processes they represent demonstrates the breadth over which the functional significance of variation can be explored using this methodology.

**FIGURE 8:**
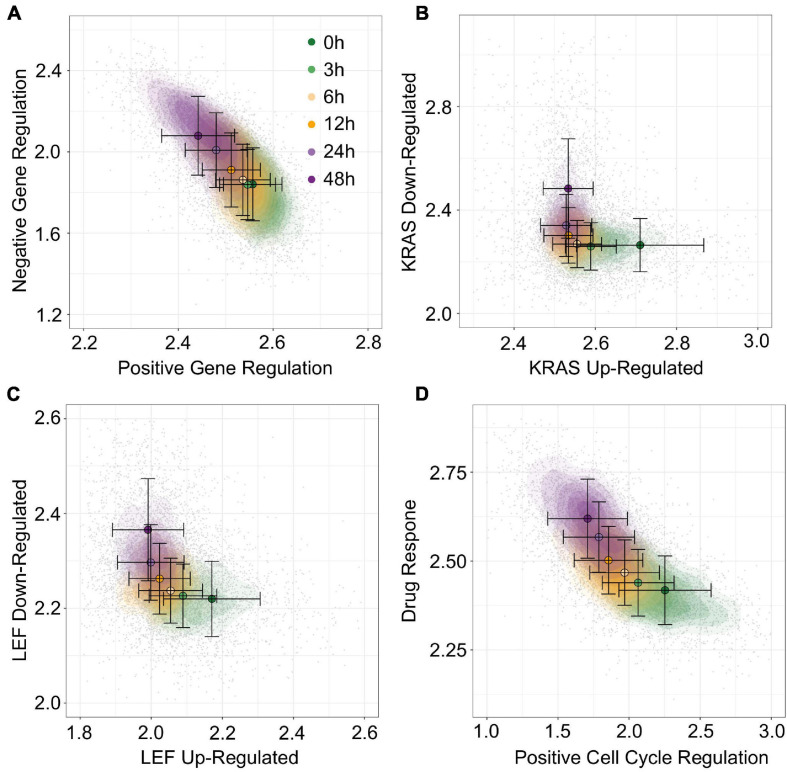
Visualization of trametinib treatment data by multiple gene sets. **(A–D)** Contour plots of trametinib treatment functional space generated using gsNMF with different gene sets: positive vs. negative gene regulation **(A)**, KRAS overexpression up and down regulation **(B)**, LEF overexpression up and down regulation **(C)**, and positive cell-cycle regulation vs. drug response **(D)**. Color indicates the time of trametinib treatment from 0 h (dark green) to 48 h (dark purple). Circles indicate the mean gene set score of samples from each time point and the associated error bars show the standard deviation.

To move beyond EMT associated data and gene sets, we next used gsNMF to analyze data from McFarland et al. (2020) which is composed of 7,245 cells with heterogeneous origins treated with trametinib for 3, 6, 12, 24, or 48 h as well as an untreated control (0 h). Because this data set mixes 24 cell lines from several different origin tissues and focuses specifically on the response to a cancer drug, we focused our exploration of functional spaces on 1,022 gene sets derived from the C6 database from Molecular Signature Database as well as the drug resistant genes identified by Wang et al. (2017) and their overlapping KEGG pathways and GO terms (see “Materials and Methods” section). Overall, 57 gene sets (5.6%) had a leading dimension whose magnitude of correlation (PCC) was >0.5 and relaxing this threshold to >0.4 yielded only 200 (19.6%) gene sets, suggesting that the explained temporal variance in this data set is lower than that obtained with A549 (Supplementary Table 5). Nevertheless, using positive regulation of gene expression (GO:0010628) and negative regulation of gene expression (GO:0010629), we were able to a functional space of trametinib response with similar performance (*R*^2^ = 0.30, Figure 8A) to our model of EMT progression in DU145 data (*R*^2^ = 0.31). Additionally, a number of oncogenic signatures which were positively correlated with trametinib response, though there were no up/down regulated pairs that with leading dimensions in opposed directions. Instead, we selected two oncogenic signatures, down regulation in response to KRAS over-expression (KRAS.600_UP.V1_DN) and down regulation in response to LEF over-expression (LEF1_UP.V1), whose leading dimension were strongly correlated with trametinib response (PCC = 0.54). We then took the negatively correlated component of the corresponding up regulation gene sets models (KRAS.600_UP.V1_UP and LEF_UP.V1_UP), even though the magnitude of the positively and negatively correlated components was similar (difference the absolute value of PCC ≤ 0.005). This process gave functional spaces which improved variance explained over the previous gene regulation model (*R*^2^ = 0.35 and 0.36, respectively, Figures 8B,C). Together, these results suggest suppression of gene expression in general and of oncogenes specifically in response to trametinib treatment, consistent with the results in McFarland et al. which observed greater enrichment of KRAS responsive genes among down-regulated genes in later time points relative to earlier ones. As with A549 data, we were also able to combine distinct functional sets, response to drug (GO:0042493, PCC = 0.58) and positive regulation of cell cycle (GO:0045787, PCC = −0.49) to explain an comparable amount of variance in expression as the reciprocal onco-gene sets (*R*^2^ = 0.36, Figure 8D). As such, while the amount of variance we can capture with our models is dependent on the data set, our approach overall is capable of producing functional spaces that broadly characterize variance in expression across diverse data and gene sets.

## Discussion

Previous methods that aimed to address the challenges of visualizing single-cell data in functional space were primarily based on weighted sum of expression values or Kolmogorov–Smirnov test with full datasets (Hänzelmann et al., 2013; DeTomaso and Yosef, 2016). These methods are useful to analyzing samples with functional gene sets, they do not provide transferability which is essential for predicting cell states with existing models and new data. We showed that constrained linear transformation enables good performance in depicting cell states with straightforward interpretation in functional space and satisfactory efficiency. While more sophisticated methods such as deep generative models have potentials to address similar problems, current methods primarily focus on the interpretability in terms of inter-sample distances in low dimensions rather than the dimensions themselves (Ding et al., 2018; Lopez et al., 2018), and we expect that the gsPCA and gsNMF methods are more efficient than models based on non-linear connectivity.

Factorization approaches like PCA and NMF have previously been applied to the problem of gene expression, with NMF in particular having been used to deconvolute expression patterns scRNA-seq data sets (Chen and Zhang, 2018; Fujita et al., 2018; Min et al., 2018; Kotliar et al., 2019; Zhang and Zhang, 2019), but these approaches have primarily focused on the unsupervised clustering of samples and/or for *de-novo* module discoveries at relatively high dimensionality (*n* > 10). In contrast, our approach suggests there is a utility in applying these factorization approaches to interrogating the relationship between known gene modules and data with implicit structure and/or separable populations of samples, particularly when assessing a single biological process (EMT) across multiple contexts (e.g., cell line, time and space), such that the simplicity of low-dimension space (*n* = 2) can be leveraged for visualization and analysis.

In this work we have found that conserved EMT gene expression signatures can be used to describe stages of EMT in multiple cell lines (e.g., A549 and DU145), and these signatures not only capture the subpopulation heterogeneity resulting from differential times of treatment with EMT-inducing signals such as TGF-β, but also reflect the EMT program driven by spatial heterogeneity with cell populations (McFaline-Figueroa et al., 2019). These results are consistent with the existence of conversed EMT program across cell lines (Cook and Vanderhyden, 2020), but do not contradict the idea of context specific expression as models trained and applied to the same data set always explained more variance in EMT progression. The coexistence of a common EMT signature and context specific expression is further supported by the observation that M-scores were more consistent and better separate data across different contexts of EMT than E-scores, and the related observation that M-gene component values were correlated across spatial and temporal models, while E-genes were not. This suggests that M-gene induction by TGF-β is consistent across cellular contexts, while changes in E-gene expression are more variable, possibly due to greater sensitivity to cell line, environmental context, or other initial conditions effecting the cell prior to induction.

The transferability of models across EMT context indicates the synergy between spatial arrangement of cells and external signals (e.g., TGF-β) in determining the stages of EMT. In addition, we found that the functional dimensions obtained with TGF-β can serve as reasonable approximations for the positioning of tumor transcriptomes in the EMT spectrum. Similar to the EMT spectrum, many biological processes involve stepwise changes of gene expression programs. A possible mechanism underlying these non-binary programs is the feedback-driven formation of stable intermediate cell states (Yui and Rothenberg, 2014; Ye et al., 2019). With the rapid advances of the single-cell technology, transcriptome-wide gene expression data will become available for more biological systems. We expect that our functional projection methods can be widely useful for visualizing and analyzing these data. In particular, the transferability of the models can be a powerful feature for interrogating the relationships among different experimental conditions and cell types.

## Materials and Methods

### Gene Expression Data Sources

Single-cell RNA-sequencing data and meta data for A549 and DU145 cell lines were obtained from Cook and Vanderhyden (2020). In brief, we obtained pre-processed SeuratObjects for A549 and DU145 TGF-β as .rds data files and extracted expression data for E, M, all genes using the ScaleData function from Seurat to regress out mitochondrial gene expression, total unique reads in a sample, cell cycle gene expression, and batch effects as well as center and scale each data set across genes (Stuart et al., 2019). For McFaline-Figueroa et al. (2019) spatial data we obtained aggregated count data from GEO in the form of a pre-processed .cds file (GSE114687). We then dropped genes expressed in less than 50 cells (∼1% of each data set) from Mock and TGFB1 and split samples into Mock and TGFB1 subset for subsequent steps. Because we planned to compare models from these data to those from A549 and DU145, we followed the preprocessing procedure from Cook and Vanderhyden: we normalized the Mock and TGFB1 data sets independently in Seurat using the NormalizeData function and then used ScaleData to regress out mitochondrial gene expression, total unique reads in a sample, and cell cycle gene expression as well as scale each data set across genes. Finally, we obtained Cell Ranger output for trametinib time-course data from McFarland et al. (2020) and processed it in R using the Read10X function. We dropped the DMSO time course, and used the Untreated samples as time 0 as well as annotations from the original manuscript to eliminate low quality cells and then filtered genes expressed in less than 73 cells (∼1% of the data set). Pre-processing was done in Seurat as with using NormalizeData and ScaleData as previously described, except that we additionally regressed out the effect of each different cell line used in the experiment, but did not regress out cell-cycle gene expression as the original manuscript suggested that cell cycle disruption may be induced by trametinib treatment.

TCGA bulk RNA-seq data was obtained from TCGAbiolinks (Colaprico et al., 2016; McFaline-Figueroa et al., 2019). Raw counts were transformed to log_2_TPM with a pseudo-count of 1 using gene models for the hg38 annotation of the human genome obtained from RefSeq (O’Leary et al., 2016).

### Non-negative PCA and NMF

Gene set non-negative principal component analysis uses the non-negative approach to PCA pioneered by Sigg and Buhmann (2008). In brief, the vector of weights, *w*, used to define the first principal component of PCA is defined such that it maximizes the variance of the first component, i.e.:

$$\arg\max_{w}w^{\text{T}}Cw$$

Where *C* is the covariance matrix of the original data set *X* and *w* is unit vector (||*w*||^2^ =  1). In our case, *X* is an *m* by *n* matrix of expression values where *m* is the number of samples and *n*is the number of genes in the selected gene set. This method for determining *w* can be treated as an expectation maximization problem where the original data is projected using the current estimate of *w* (*y* = *Xw*_*t*_) and this projection is used to re-estimate *w* using the following minimization step:

$$w_{t+1}=\arg\min_{w}\sum_{n=1}^{N}{||x_{n}-y_{n}w||}_{2}^{2}$$

Where *x*_*n*_ are the rows of the original data and *y*_*n*_ are the rows of the projected data (Sigg and Buhmann, 2008). This expectation-maximization formulation allows additional constraints on *w*, including forcing the component values to be non-negative. Note that the non-negativity constraint applies only to the weight components such that negative scores can still exist if there are negative values in underlying data, such as those produced by centering expression data to zero which we did for all gsPCA inputs. Subsequent components are calculated in the same way, under the constraint that they are orthogonal to the preceding ones.

NMF involves factorizing the original data matrix of non-negative values into two matrices whose product estimates the original data, i.e.:

X≅WH

Where *X* is the original matrix (*m* by *n*)**, *W*** is the basis or features matrix (*m* by *p*), and *H* is the coefficient matrix (*p* by *n*), such that *m* is the number of rows in the original matrix (samples in our case), *n* is the columns (genes in our case), and *p* is the number of components used in the factorization. In addition to factorizing *X*, NMF naturally clusters the elements of the original data: *W* represents the “centers” of column clusters whose memberships is determined by the relative coefficient values in *H*, and vice versa with *H* representing the centers of row clusters determined by *W* (Brunet et al., 2004). Because the original matrix is constrained to being non-negative, we subtracted the minimum of value of the scaled expression matrix from all values to create a non-negative input matrix. As a consequence, the values of the *W* and *H* matrices must likewise be non-negative such that product is non-negative.

### Implementation of Dimension Reduction Approaches

We implemented non-negative PCA in R using the *nsprcomp* function (with the option nneg = TRUE) from the package of the same name (Sigg and Buhmann, 2008). We used the standard convergence parameters for the algorithm as these produced consistent principal components across multiple runs and different number of components. This is to be expected as *nsprcomp* greedily maximizes the variation explained by each component in order. For gsNMF, we used the Scikit-learn implementation of NMF (Pedregosa et al., 2011). To optimize convergence criteria, we performed a cross-validation analysis of A549 data and found that a two-component model fit with a tolerance of 1e–6 and a max of 500 iterations gave the best results (see Supplemental Methods and Supplementary Figure 6). We also tested ten random seeds of the two component A549 model on the full data to confirm that consistent results were given (average PCC of dimensions > 0.99). We tested ten random seeds against the other data sets to tune the convergence parameters, raising maximum iterations to 2,500 and tolerance to 1e–9 if the initial parameters did not yield consistent results (i.e., average PCC of dimensions > 0.99). GSVA and Z-score methods were implemented using the GSVA package in R (Hänzelmann et al., 2013).

Unlike GSVA and Z-scores methods, which produce a single score per gene set, gsPCA and gsNMF both produce multiple sample level scores in the form of principal component scores (*wX*, gsPCA) or the columns of the features matrix (*W*, gsNMF), while the corresponding loading values/weights (*w*, gsPCA) or coefficient matrix (*H*, gsNMF) represent gene level scores (i.e., gene importance). Therefore, we need to choose one of these sample level scores as a “leading dimension” to represent each gene set in functional space. For gsPCA, we used the first principal component as this represents the direction of greatest variance for gene expression in that gene set. For gsNMF, we used the magnitude of correlation between the columns of the transform matrix and the sample metric that best represented progression in EMT (i.e., time for A549 and DU145 data). For E and M genes, we also required the sign of correlation to match the expected change in E and M genes during EMT (i.e., picking the greatest negative PCC for E and the greatest positive PCC for M). For our spatial EMT data, where there were only two populations, *f* probability was used instead (see below), but with the same constraint on the direction of E and M dimensions (i.e., higher M scores for outer samples and higher E scores for inner samples).

### Evaluating Functional Spaces

To evaluate a functional space, we used two metrics. First, if the sample data had an associated time variable, we created a model of time as a linear function of the two axes of the functional space (time ∼X + Y) and calculated the coefficient of determination, which is the percent of overall variance in the dependent variable explained by the independent variables (Adjusted *R*^2^). Second, to evaluate the ability of functional space to separate distinct populations, such as neighboring time points or spatial locations, we used the common language effect size (*f*), which is the probability that a value or score randomly sampled from one population will be larger than a random score from the other. This metric is advantageous because we can calculate it from the test statistic of Mann–Whitney *U*-test, which also provide a measure of significance, and is related to the area under of the receiver operating curve (AUC-ROC), which is commonly used to asses classification algorithms. Additionally, since the *f* probability is reciprocal, the choice which population we want to be larger is arbitrary, so for EMT we can chose to calculate the *f* probability such that the larger population is the more progressed for M and less progressed for E. Therefore, a higher probability of *f* always indicates better correspondence with EMT progression in our results.

### Inference and Model Transfer

To infer the position of new data in functional space for gsPCA, we multiplied the new data directly by loading vectors (also known as weights, *w*) of the E- and M-scores. For gsNMF, we used the Scikit-learn “transform” method which transforms the input data according to the fitted model (i.e., it fixes the coefficient matrix, *H*, and generates a new feature matrix, *W*). In both cases, we used the same leading dimension for inference as in the original model. For inferring missing data points, no further steps were required as the new data always had the same coverage of the E and M gene sets as the original. However, for transferring models across cell-line, TCGA, and spatial data, we first had to determine the common set of genes between the two data sets. Common genes were then used to filter the weight vectors for gsPCA and to refit the model on the original data using the common subset of genes for gsNMF. The data set that was the target of the transferred model was then subset by the same common set of genes and inference was done as described previously. Transferred models were assessed against the new data set using the same approaches as the original models, but relationship between E/M-scores and gene loading/coefficient values between models were assessed by PCC.

### Multi-Gene Set Evaluation

C2 gene sets were obtained from the Molecular Signature Database (version 7.1)^1^ (Subramanian et al., 2005; Liberzon et al., 2011, 2015). gsNMF was performed as described for EMT gene sets expect that we increased the iteration (2,500) and convergence threshold (1e–9) of the NMF algorithm to ensure consistent results across the gene sets which varied widely in size (2–1,581 genes present in the data set) and coverage by the A549 data set due to the sparsity of scRNA-seq data. To test the robustness of this approach, we looked at the correlation of PCC scores along the leading axis for each gene set across 10 random seeds and found they were highly similar (average PCC between seeds = 0.998).

We used the same iteration and convergence threshold for analysis of the C6 (Molecular Signature Database) and the GEAR drug resistance gene sets (Wang et al., 2017) which were used to project the trametinib data. Gene sets, KEGG pathways and GO terms associated with the GEAR drug genes were obtained using KEGGREST package in R for KEGG pathways and http://geneontology.org/ for GO terms (Ashburner et al., 2000; The Gene Ontology Consortium, 2017).

## Data Availability Statement

Code and data for generating the primarily results of this study can be found at https://github.com/panchyni/gsNMF.

## Author Contributions

TH designed the research. NP and TH performed the research and wrote the manuscript. NP, KW, and TH analyzed the data. All authors contributed to the article and approved the submitted version.

## Conflict of Interest

The authors declare that the research was conducted in the absence of any commercial or financial relationships that could be construed as a potential conflict of interest.

## Publisher’s Note

All claims expressed in this article are solely those of the authors and do not necessarily represent those of their affiliated organizations, or those of the publisher, the editors and the reviewers. Any product that may be evaluated in this article, or claim that may be made by its manufacturer, is not guaranteed or endorsed by the publisher.

## Footnotes

1
http://www.gsea-msigdb.org/gsea/msigdb/index.jsp

## Supplementary Material

Supplementary Figure 1 Predicting A549 samples from specific time points using gsPCA. **(A)** Scatter plot of E (X-axis) and M (Y-axis) scores for all TGF-β induction samples using gsPCA. Samples from different time points are indicated by color going from 0 days (dark green) to 7 days (dark purple). **(B–D)** Scatter plot of 0-day (green, **B**), 1-day (yellow, **C**), and 7-day samples (purple, **D**) inferred using a gsPCA model built using all other time points (gray). **(E)** A scatter plot of TGF-β induction samples with TGF-β reversion samples (i.e., 7 days induction followed by removal from TGF-β). Induction samples are labeled as in panel **(A)**, while reversion samples are colored blue, with darker shade indicating longer time since removal. **(F)** Scatter plot of 3-day reversion samples (dark blue) inferred using a gsPCA model built using all non-reversion time points (gray).

Supplementary Figure 2 Transferring gsPCA models between A549 and DU145 TGF-β induced samples. **(A–D)** Scatter plot of E (X-axis) and M (Y-axis) scores for different combinations of data and gsPCA model: **(A)** A549 model on A549 data, **(B)** DU145 model on A549 data, **(C)** A549 model on DU145 data, and **(D)** DU145 model on DU145 data. Samples from different time points are indicated by color going from 0 days (dark green) to 7 days (dark purple). **(E,F)** Comparison of E-scores of samples from A549 **(E)** and DU145 **(F)** data. The X-axis is the E-score from using the model from the same data set (A549 on A549 and DU145 by DU145), while the Y-axis is the E-score from the opposite model (DU145 on A549 and A549 on DU145). Samples from different time points are indicated by color going from 0 days (dark green) to 7 days (dark purple). **(G,H)** Comparison of M-scores of samples from A549 **(G)** and DU145 **(H)** data. The X-axis is the M-score from using the model from the same data set (A549 on A549 and DU145 by DU145), while the Y-axis is the M-score from the opposite model (DU145 on A549 and A549 on DU145). Samples from different time points are indicated by color going from 0 days (dark green) to 7 days (dark purple).

Supplementary Figure 3 Transferring gsPCA models to TCGA data. **(A,B)** Scatter plots of E-scores for PRAD **(A)** and LUAD **(B)** from transferring gsPCA models built on A549 (X-axis) and DU145 (Y-axis) data. The color of individual points indicates the original GSVA based E-score of the TCGA data set. **(C,D)** Scatter plots of M-scores for PRAD **(C)** and LUAD **(D)** from transferring gsPCA models built on A549 (X-axis) and DU145 (Y-axis) data. The color of individual points indicates the original GSVA based M-score of the TCGA data set.

Supplementary Figure 4 Transferring gsPCA models between temporal and spatial data sets. **(A–C)** Scatter plots of E (X-axis) and M (Y-axis) scores for Mock spatial data from gsPCA models built on different data sets: Mock spatial data **(A)**, TGF-β induced spatial data **(B)**, and TGF-β induced A549 temporal data **(C)**. The color of the sample indicates whether it originates from a cell in the inner-ring (non-motile, red) or the outer ring (motile, blue). **(D–F)** Scatter plots of E (X-axis) and M (Y-axis) scores for TGF-β spatial data from gsPCA models built on different data sets: TGF-β induced spatial data **(D)**, Mock spatial data **(E)**, and TGF-β induced A549 temporal data **(F)**. The color of the sample indicates whether it originates from a cell in the inner-ring (non-motile, red) or the outer ring (motile, blue).

Supplementary Figure 5 Normalized expression of KRT8 and KRT18 across A549 and migration data sets. Boxplots showing the normalized expression of KRT8 **(top)** and KRT18 **(bottom)** across A549 data **(left)** and migration data **(right)**. The central black line indicates the average of each distribution while the whiskers show 1.5 times the interquartile range. For A549 data, color of the boxplot indicates the time from 0 day (dark green) to 7 days (dark purple) for TGF-β treatment, followed by removal of TGF-β for 8 h, 1 days, and 3 days (darkening shades of blue). For migration data, color differentiates samples in the inner (red) vs. outer (blue) rings of the assay.

Supplementary Figure 6 Performance of gsPCA models across cross-validation data sets. Boxplots showing Adjusted *R*^2^ of the linear model of gsNMF leading E and M dimensions across different number of model components (X-axis) and different convergence criteria: basic (algorithm standard, **A**), strong (1e–6 tolerance, 500 iterations, **B**), very strong (1e–9 tolerance, 2,500 iterations, **C**), and nndsvd (initialization with non-negative singular value decomposition, 1e–6 tolerance, 500 iterations, **D**). Left and right panels separate the result for all validation folds and the mean performance of independent folds within each training data set (see Supplemental Methods). The yellow line indicates the average of each distribution while the whiskers show 1.5 times the interquartile range. Red dots show the individual Adjusted *R*^2^ values in each distribution.
