# Supplementary figures and images for: Interpretable, Scalable, and Transferrable Functional Projection of Large-Scale Transcriptome Data Using Constrained Matrix Decomposition
Source: Front Genet. 2021 Aug 20;12:719099. doi: 10.3389/fgene.2021.719099 (PMC8417714; doi:10.3389/fgene.2021.719099)

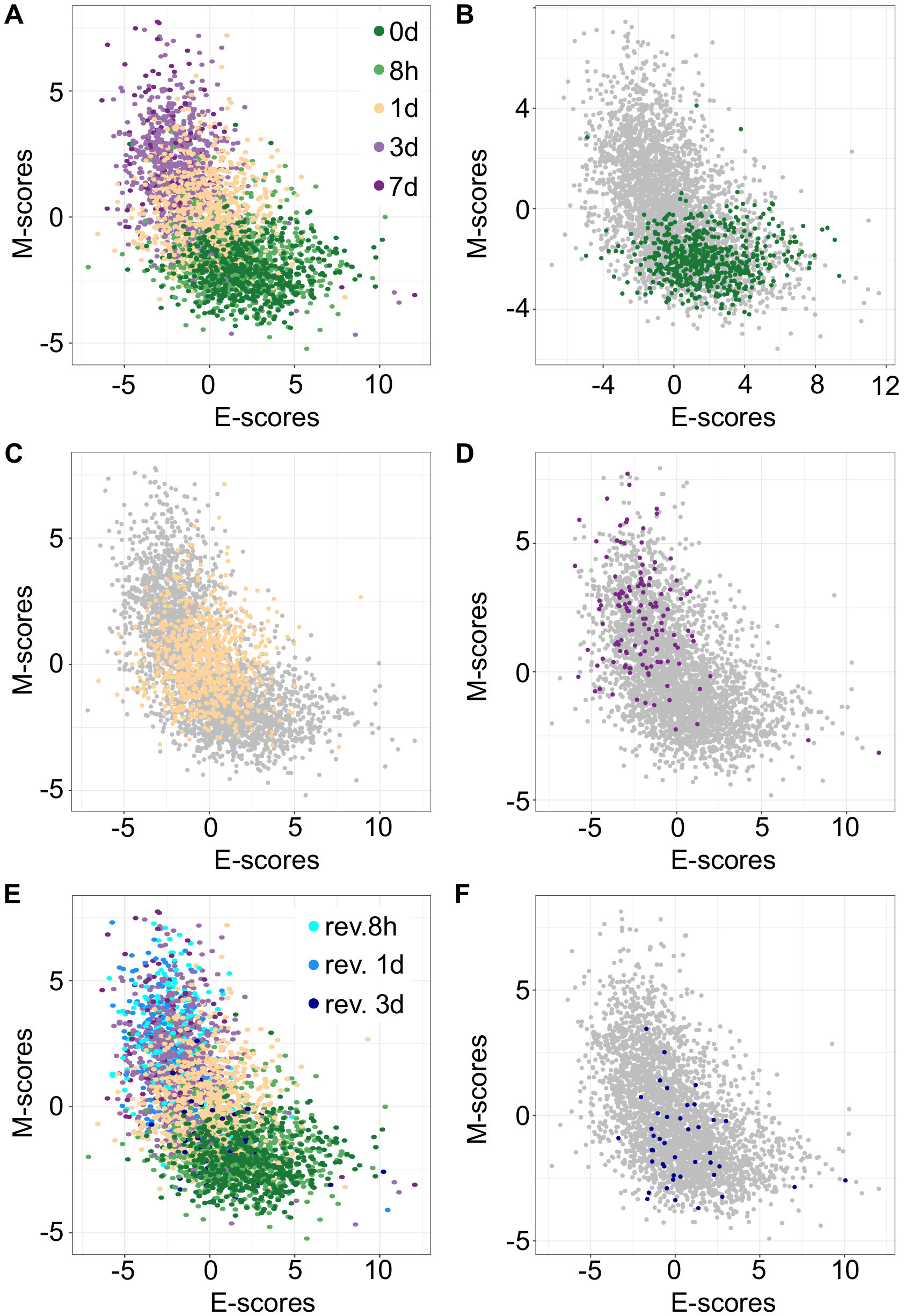

Supplement: Supplementary Figure 1 — Predicting A549 samples from specific time points using gsPCA. (A) Scatter plot of E (X-axis) and M (Y-axis) scores for all TGF-β induction samples using gsPCA. Samples from different time points are indicated by color going from 0 days (dark green) to 7 days (dark purple). (B–D) Scatter plot of 0-day (green, B), 1-day (yellow, C), and 7-day samples (purple, D) inferred using a gsPCA model built using all other time points (gray). (E) A scatter plot of TGF-β induction samples with TGF-β reversion samples (i.e., 7 days induction followed by removal from TGF-β). Induction samples are labeled as in panel (A), while reversion samples are colored blue, with darker shade indicating longer time since removal. (F) Scatter plot of 3-day reversion samples (dark blue) inferred using a gsPCA model built using all non-reversion time points (gray). [file Image_1.JPEG]

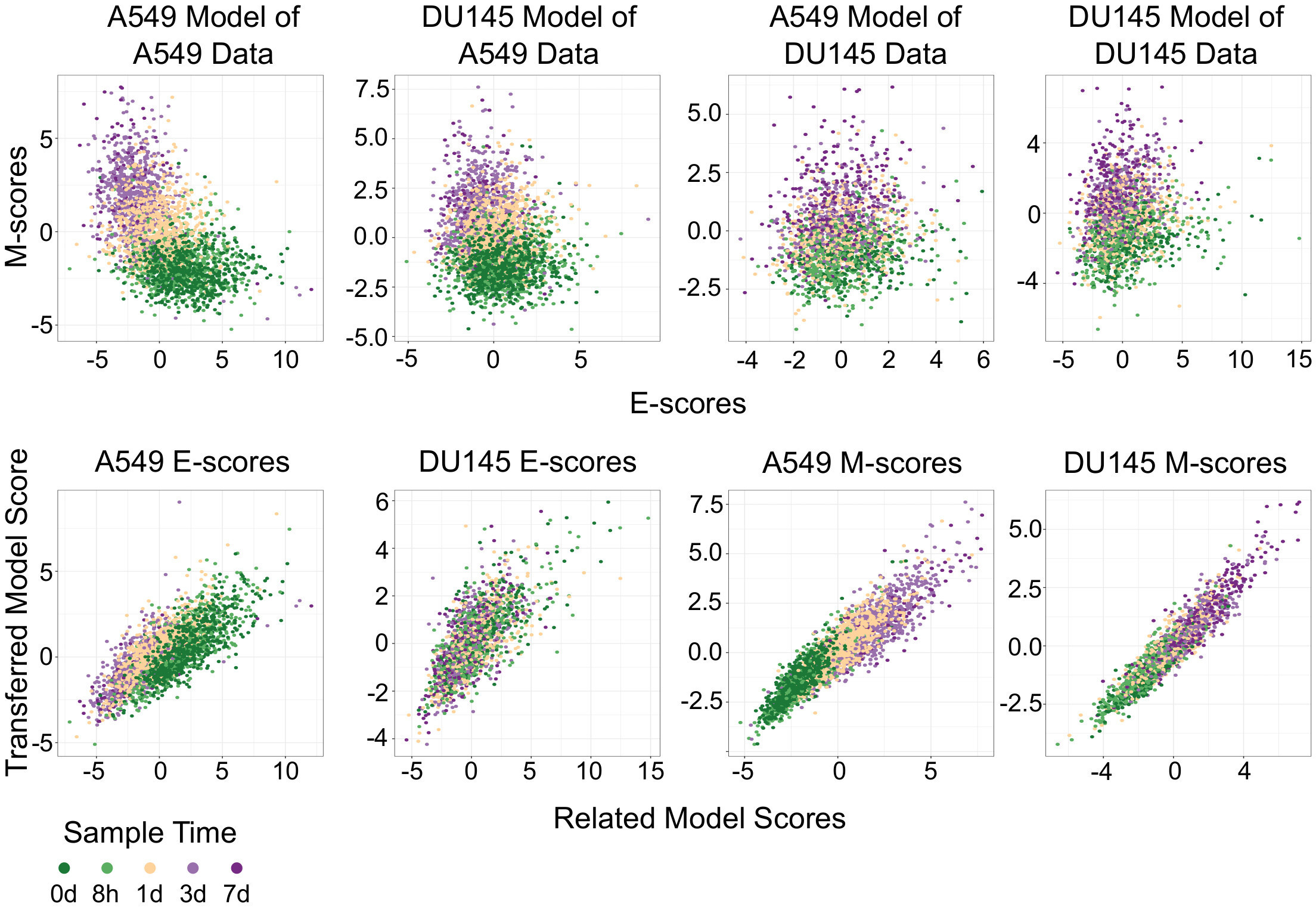

Supplement: Supplementary Figure 2 — Transferring gsPCA models between A549 and DU145 TGF-β induced samples. (A–D) Scatter plot of E (X-axis) and M (Y-axis) scores for different combinations of data and gsPCA model: (A) A549 model on A549 data, (B) DU145 model on A549 data, (C) A549 model on DU145 data, and (D) DU145 model on DU145 data. Samples from different time points are indicated by color going from 0 days (dark green) to 7 days (dark purple). (E,F) Comparison of E-scores of samples from A549 (E) and DU145 (F) data. The X-axis is the E-score from using the model from the same data set (A549 on A549 and DU145 by DU145), while the Y-axis is the E-score from the opposite model (DU145 on A549 and A549 on DU145). Samples from different time points are indicated by color going from 0 days (dark green) to 7 days (dark purple). (G,H) Comparison of M-scores of samples from A549 (G) and DU145 (H) data. The X-axis is the M-score from using the model from the same data set (A549 on A549 and DU145 by DU145), while the Y-axis is the M-score from the opposite model (DU145 on A549 and A549 on DU145). Samples from different time points are indicated by color going from 0 days (dark green) to 7 days (dark purple). [file Image_2.JPEG]

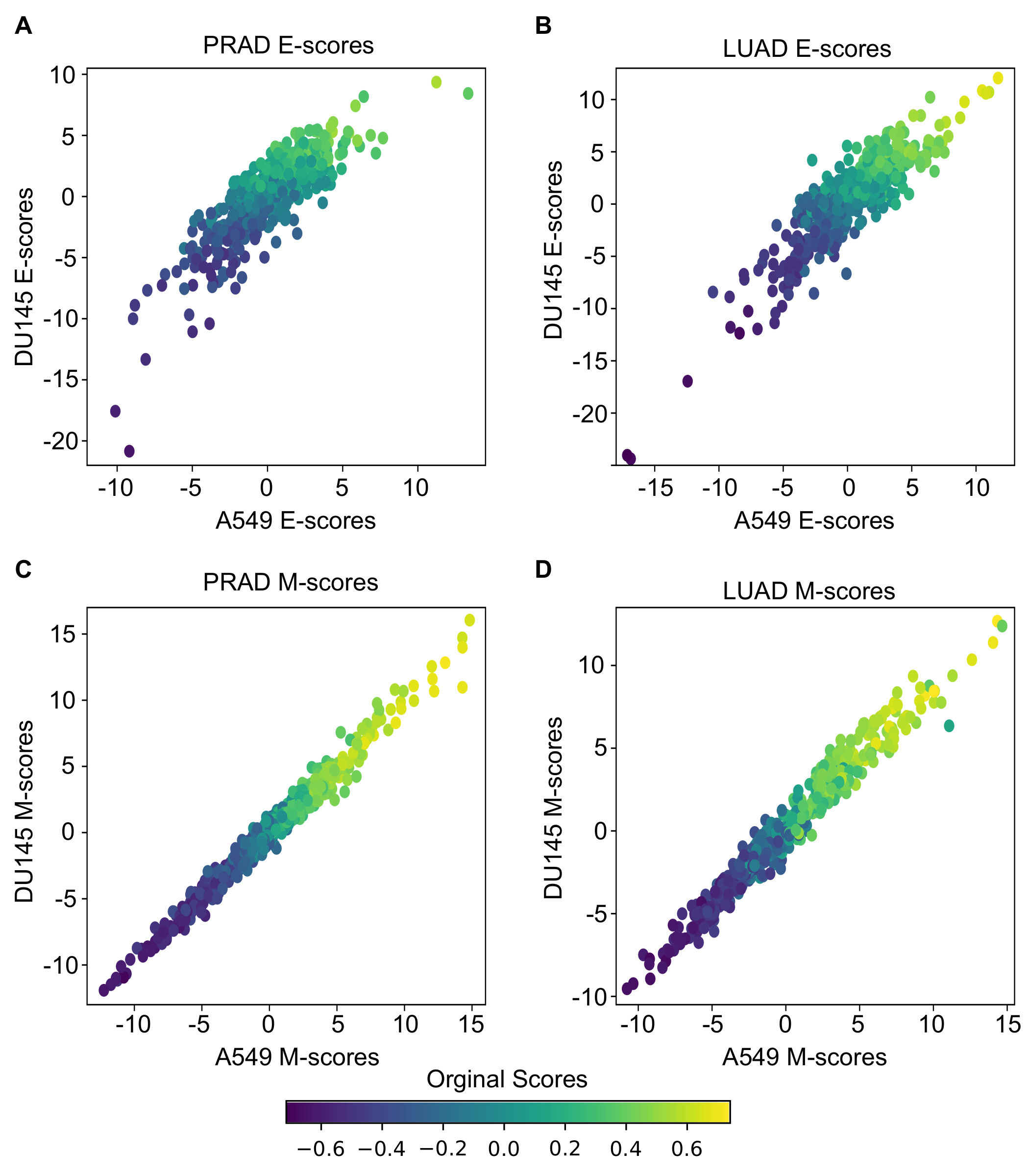

Supplement: Supplementary Figure 3 — Transferring gsPCA models to TCGA data. (A,B) Scatter plots of E-scores for PRAD (A) and LUAD (B) from transferring gsPCA models built on A549 (X-axis) and DU145 (Y-axis) data. The color of individual points indicates the original GSVA based E-score of the TCGA data set. (C,D) Scatter plots of M-scores for PRAD (C) and LUAD (D) from transferring gsPCA models built on A549 (X-axis) and DU145 (Y-axis) data. The color of individual points indicates the original GSVA based M-score of the TCGA data set. [file Image_3.JPEG]

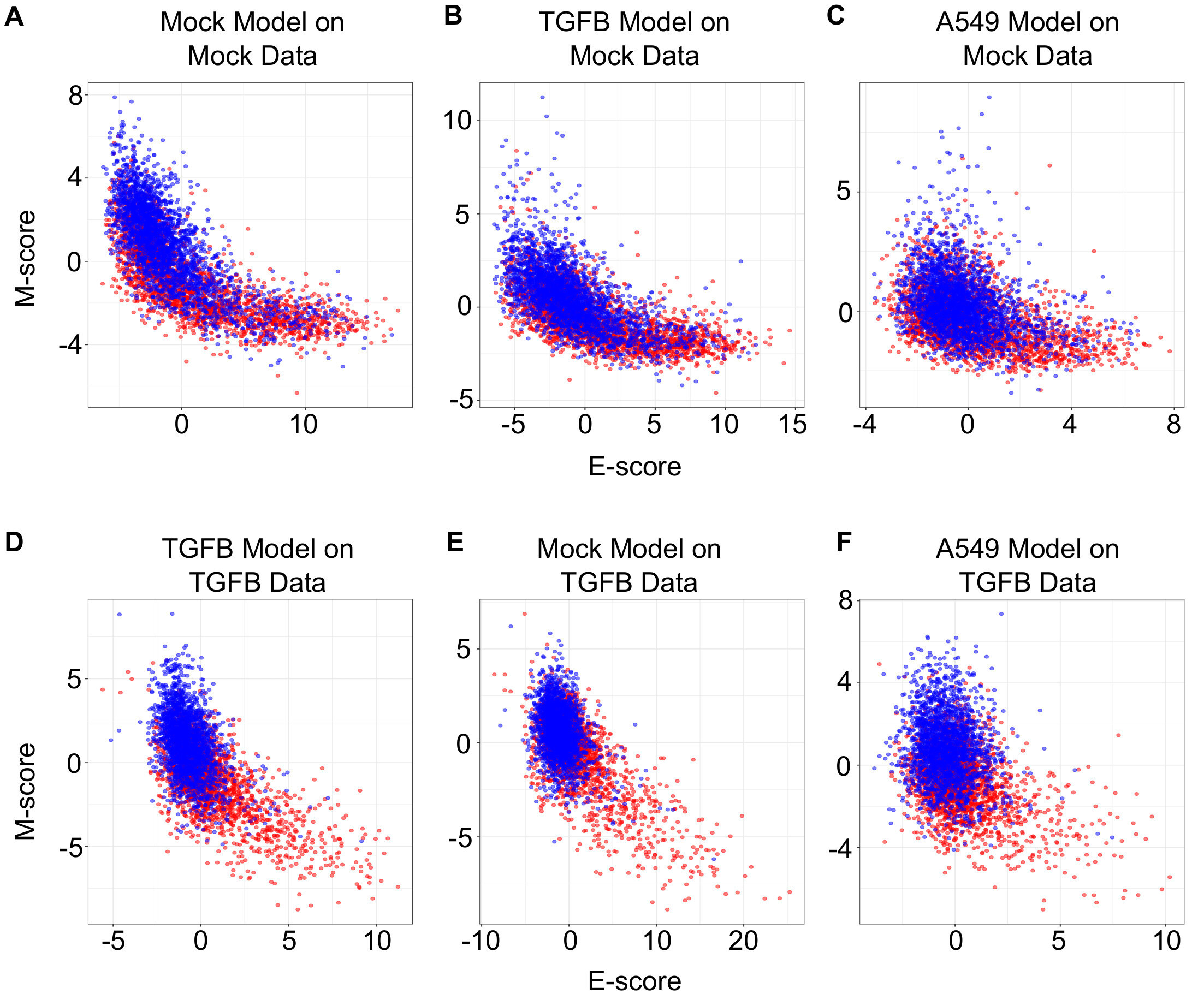

Supplement: Supplementary Figure 4 — Transferring gsPCA models between temporal and spatial data sets. (A–C) Scatter plots of E (X-axis) and M (Y-axis) scores for Mock spatial data from gsPCA models built on different data sets: Mock spatial data (A), TGF-β induced spatial data (B), and TGF-β induced A549 temporal data (C). The color of the sample indicates whether it originates from a cell in the inner-ring (non-motile, red) or the outer ring (motile, blue). (D–F) Scatter plots of E (X-axis) and M (Y-axis) scores for TGF-β spatial data from gsPCA models built on different data sets: TGF-β induced spatial data (D), Mock spatial data (E), and TGF-β induced A549 temporal data (F). The color of the sample indicates whether it originates from a cell in the inner-ring (non-motile, red) or the outer ring (motile, blue). [file Image_4.JPEG]

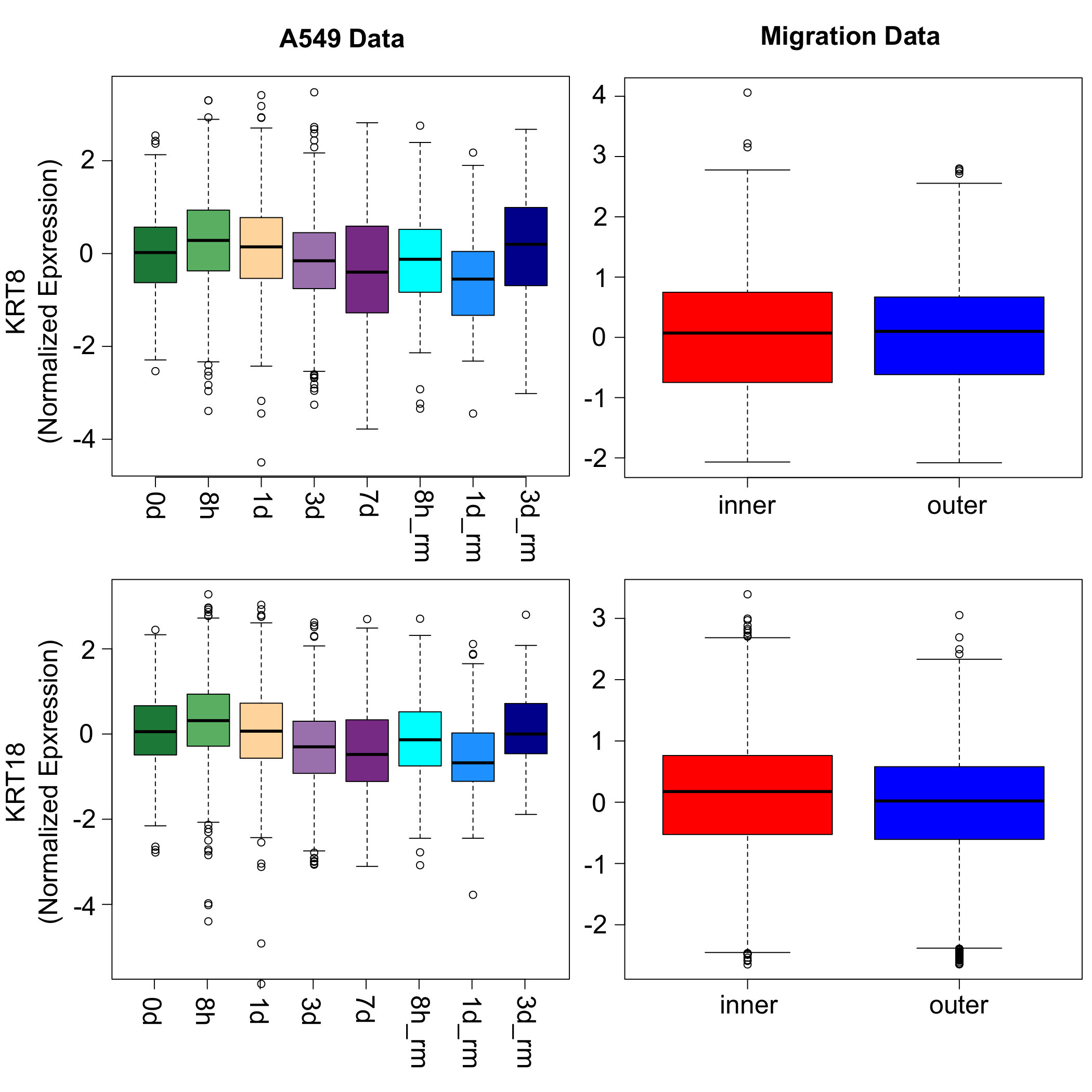

Supplement: Supplementary Figure 5 — Normalized expression of KRT8 and KRT18 across A549 and migration data sets. Boxplots showing the normalized expression of KRT8 (top) and KRT18 (bottom) across A549 data (left) and migration data (right). The central black line indicates the average of each distribution while the whiskers show 1.5 times the interquartile range. For A549 data, color of the boxplot indicates the time from 0 day (dark green) to 7 days (dark purple) for TGF-β treatment, followed by removal of TGF-β for 8 h, 1 days, and 3 days (darkening shades of blue). For migration data, color differentiates samples in the inner (red) vs. outer (blue) rings of the assay. [file Image_5.JPEG]

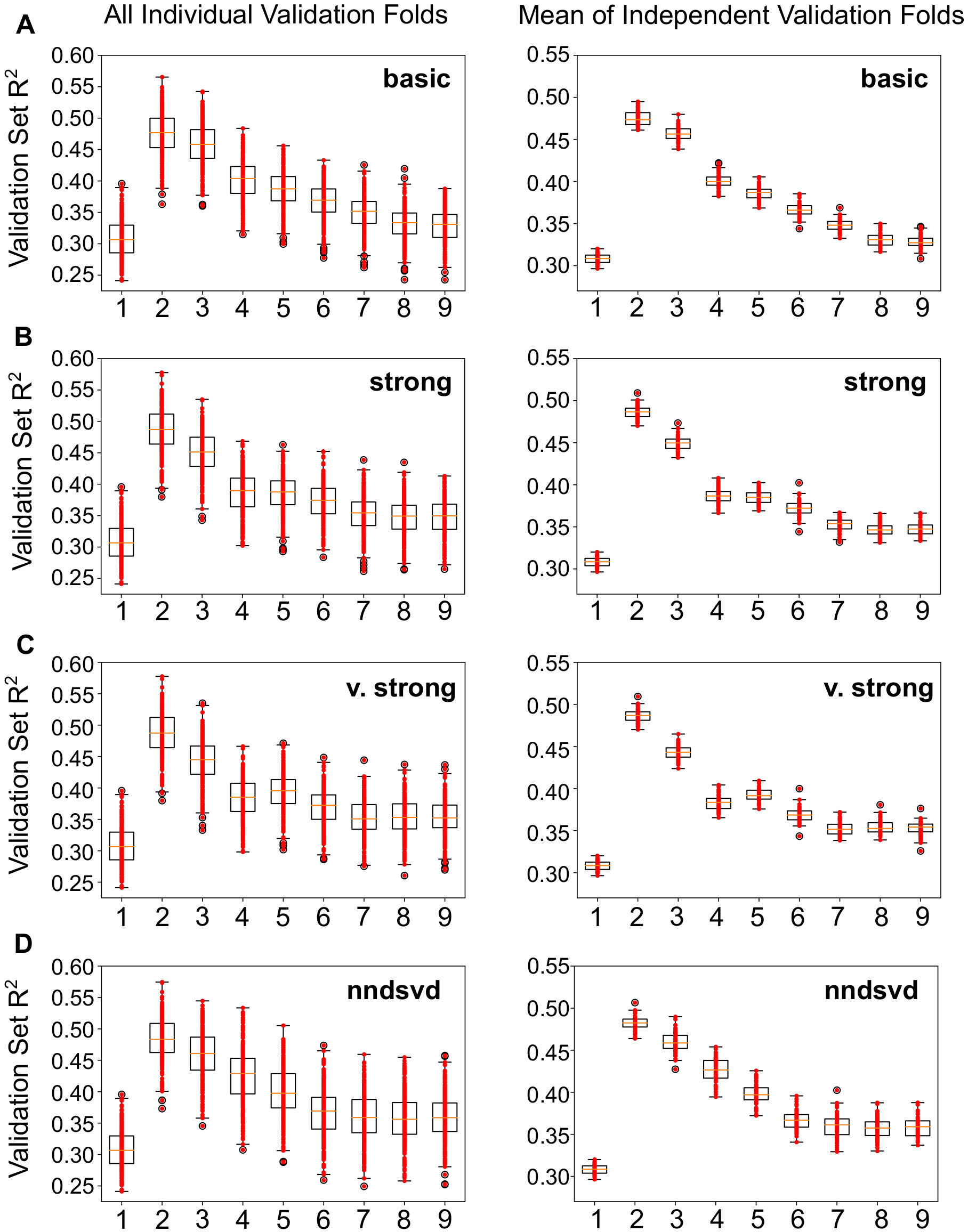

Supplement: Supplementary Figure 6 — Performance of gsPCA models across cross-validation data sets. Boxplots showing Adjusted R2 of the linear model of gsNMF leading E and M dimensions across different number of model components (X-axis) and different convergence criteria: basic (algorithm standard, A), strong (1e–6 tolerance, 500 iterations, B), very strong (1e–9 tolerance, 2,500 iterations, C), and nndsvd (initialization with non-negative singular value decomposition, 1e–6 tolerance, 500 iterations, D). Left and right panels separate the result for all validation folds and the mean performance of independent folds within each training data set (see Supplemental Methods). The yellow line indicates the average of each distribution while the whiskers show 1.5 times the interquartile range. Red dots show the individual Adjusted R2 values in each distribution. [file Image_6.JPEG]
